# Comparison of Supraclavicular Oblique Incision With Traditional Low Collar Incision Approach for Thyroidectomy in Differentiated Thyroid Cancer

**DOI:** 10.3389/fonc.2022.842981

**Published:** 2022-03-15

**Authors:** Bo Jiang, Cheng Qu, Chaoyu Jiang, Chen Zhang, Song Shen, Yuqian Luo, Lei Su

**Affiliations:** ^1^ Department of General Surgery, Nanjing Drum Tower Hospital, Clinical College of Nanjing Medical University, Nanjing, China; ^2^ Department of General Surgery, Affiliated Drum Tower Hospital, Medical School of Nanjing University, Nanjing, China; ^3^ Department of Cardiology, Nanjing Drum Tower Hospital, Medical School of Nanjing University, Nanjing, China; ^4^ Department of Laboratory Medicine, Nanjing Drum Tower Hospital, Medical School of Nanjing University, Nanjing, China

**Keywords:** supraclavicular oblique incision thyroidectomy, traditional low collar incision thyroidectomy, differentiated thyroid cancers, minimally invasive surgery, retrospective study

## Abstract

**Background:**

Various incisions and approaches for thyroidectomy have been developed to treat differentiated thyroid cancer (DTC). Supraclavicular oblique incision (SOI) thyroidectomy (SOIT) has been applied in DTC patients over the past ten years. However, the safety and efficacy of this approach were yet to be confirmed.

**Aim:**

This study aimed to compare the surgical and patient-related outcomes between SOIT and traditional low collar incision thyroidectomy (TLCIT) in patients with DTC.

**Methods:**

We retrospectively screened all patients with DTC who received thyroid lobectomy from October 2020 to October 2021. The surgical results and patient-related outcomes assessed at 1 and 6 months after surgery by questionnaire were compared between the SOIT and TLCIT groups.

**Results:**

A total of 128 patients were included in this study, of whom 38 patients (30.5%) were operated on with SOIT and 89 patients (69.5%) with TLCIT. There was no significant difference in demographic characteristics and thyroid features between the two groups. Despite comparable operative time (61.9 ± 12.1 vs. 59.9 ± 15.0 min, p = 0.425), the SOIT group had a smaller neck incision (4.4 ± 0.7 vs. 5.0 ± 1.0 cm, p = 0.002), a shorter duration of postoperative drainage (2.4 ± 0.5 vs. 2.7 ± 0.9 days, p = 0.019), less volume of postoperative drainage (48.4 ± 24.6 vs. 60.3 ± 22.8 ml, p = 0.040), and shorter postoperative hospitalization (3.2 ± 0.5 vs. 3.6 ± 0.9 days p = 0.006), as compared with the TLCIT group. At 1-month follow-up after surgery, SOIT showed better performance in preventing hypoparathyroidism (p = 0.026) and abnormal neck sensation (p = 0.010) and in improving cosmetic satisfaction (p = 0.036) than TLCIT. At 6-month follow-up, SOIT was feedback with better cosmetic satisfaction (p < 0.001) and a lower percent of abnormal neck sensation (p = 0.031) or movement (p = 0.005).

**Conclusion:**

Our study suggests that minimally invasive surgery using the SOI provides superior surgical and patient-related outcomes compared with surgery using a traditional low collar incision (TLCI) in patients with DTC.

## Introduction

Thyroid cancer is one of the most common endocrine malignancies with an increasing incidence worldwide over the past two decades (1, 2). According to the Chinese cancer statistics in 2018, thyroid cancer is the 4th most common malignancy in women, accounting for approximately 4.46% of all malignancies in China (3). In thyroid cancer, differentiated thyroid cancers (DTCs), including papillary thyroid carcinoma (PTC) and follicular thyroid carcinoma (FTC), are the most prevalent histological types with a male-to-female ratio of 1:4 (4, 5).

Traditional low collar incision (TLCI) is the conventional surgical approach of thyroidectomy for treating DTCs. However, the following shortcomings of this approach are found in practice: 1) the surgery space is limited, making it challenging to dissect the superior thyroid vessels, the recurrent laryngeal nerve, and the superior parathyroid glands. For this reason, the anterior cervical band is cut sometimes to expose the surgical area more extensively. 2) Traditional surgical approach causes great damage to the neck tissues, leading to postoperative neck tissue edema and scar growth on the anterior neck. 3) Postoperative scars formed on the front of the middle neck (usually 6–8 cm in length) can cause serious aesthetic problems. Therefore, more effective and aesthetically satisfying procedures have been attempted.

The supraclavicular oblique incision (SOI) is a unilateral 2- to 3-cm incision above the clavicle along the lateral border, which was developed to achieve a better esthetic result over the past decade (6, 7). The postoperative scar resulting from such an incision is less remarkable and more easily to be concealed than that from TLCI. However, the efficacy and long-term outcomes of the approach with SOI in thyroid lobectomy were poorly studied. This study aimed to compare perioperative and long-time outcomes of the TLCI thyroidectomy (TLCIT) with the SOI thyroidectomy (SOIT) in patients with DTC.

## Methods

### Study Design and Participants

This study retrospectively screened all DTC patients receiving thyroid lobectomy admitted to the Department of General Surgery, The Affiliated Drum Tower Hospital of Nanjing University Medical School, from October 2020 to October 2021. The exclusion criterion was as follows: 1) receiving therapeutic lateral neck dissection; 2) with other pathological types of thyroid cancer; 3) with distant metastasis; 4) with recurrent tumor; and 5) with a history of neck irradiation or previous neck surgery.

### Data Collection

All the data were extracted from a thyroid database (TDatabase) at the Affiliated Drum Tower Hospital of Nanjing University Medical School, which were collected prospectively. This study was conducted in accordance with the Declaration of Helsinki (as revised in 2013) and was approved by the institutional review board (No. 2021 GLTDMC-008). Routine written informed consent was obtained for data collection, storage, and academic use of data from all patients or next of kin at admission. Additional informed consent from individuals was waived due to the retrospective and anonymous nature of the current study.

The demographic characteristics (including age and gender), thyroid tumor features (including tumor size, tumor location, and TNM stage), thyroidectomy method, and clinical outcomes of each patient were collected and recorded. Patients were assessed with thyroid function tests during the first 24 h after admission. All the laboratory results were obtained from the Central Laboratory of Nanjing Drum Tower Hospital according to the standard protocols.

### The Procedure of Supraclavicular Oblique Incision Thyroidectomy

**Figure 1:**
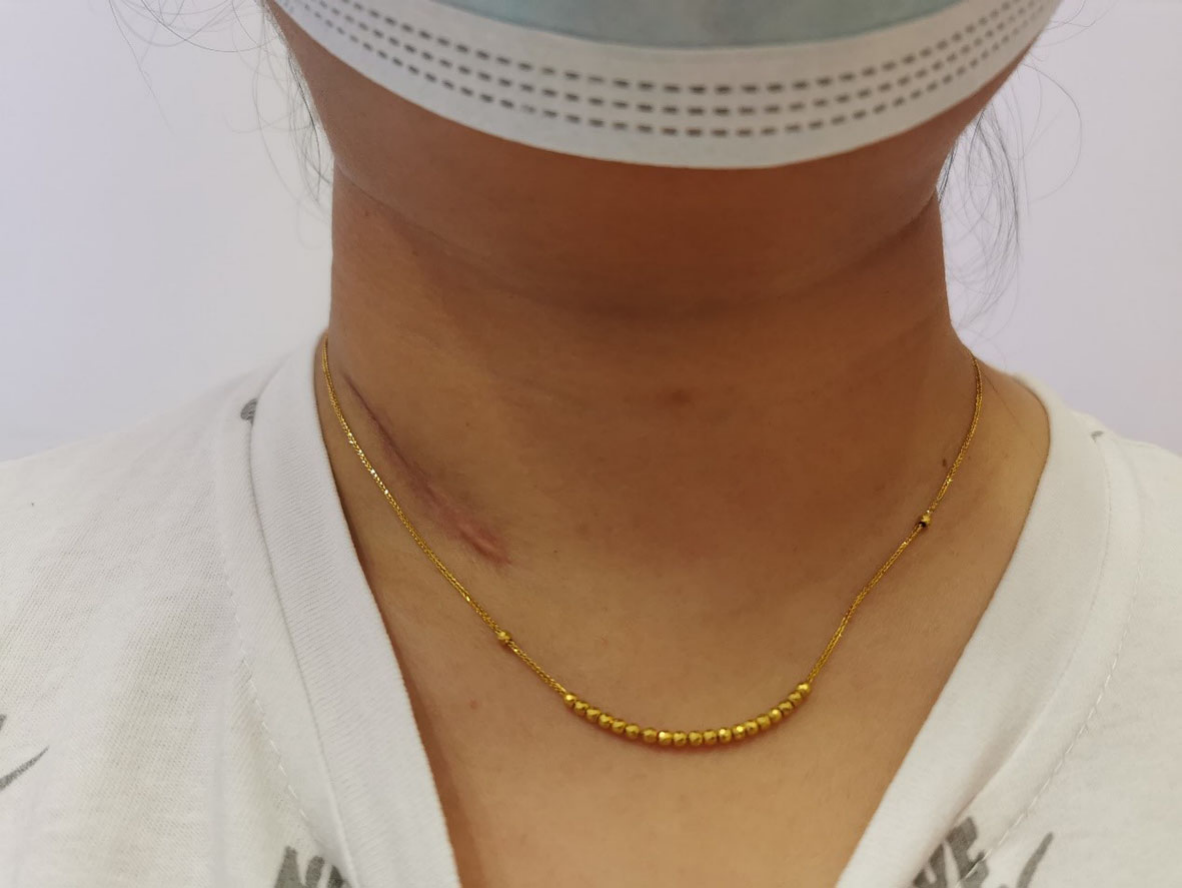
The appearance of the incision 1 month after the operation is demonstrated.

All operations were performed according to a standard protocol. The patient was endotracheal intubated with general anesthesia and placed in the supine position. The neck was extended, and the head was inclined to the healthy side to expose the surgical site. The 2- to 3-cm incision between the front edge of the sternocleidomastoid muscle and the external jugular vein was made above the clavicle along the skin crease in the lesion side (as seen in **Figure 1**). After dividing the platysma with monopolar electrocautery, the operator developed the subplatysmal space upwards to the thyroid cartilage plane and down to the upper sternal notch. The cervical fascia was incised along the front edge of the sternocleidomastoid muscle without damaging the internal jugular vein. The thyroid compartment was accessed between the sternohyoid muscles. After the thyroid gland was exposed and lifted, the middle thyroid vein and superior and inferior pole blood vessels were divided and ligated using an energetic surgical instrument. After the inferior parathyroid gland and the recurrent laryngeal nerve were confirmed and preserved, the ipsilateral thyroid lobe and the isthmus were then dissected. Then ipsilateral prophylactic central neck dissection was performed routinely. A drainage catheter was placed in the compartment of the removed thyroid lobe.

### The Procedure of Traditional Low Collar Incision Thyroidectomy

The patient was placed in the supine position neck hyperextension. A 5- to 7-cm arc incision was made along the dermatoglyphic direction, 1–2 cm above the sternal notch. After the anterior cervical band was separated *via* the white line of the neck, the thyroid gland was exposed by pulling on the anterior neck muscles. The dissection of the thyroid lobe, isthmus, and ipsilateral central cervical lymph node (LN) was performed in the same way as SOIT.

### Management After Surgery and Long-Time Follow-Up

All patients received standardized management based on the ATA management guidelines (2015) after surgery. This included pain control, thyroid-stimulating hormone (TSH) suppression therapy, and reducing vocal fold edema. The neck catheter drains were audited every day and removed when the volume was less than 10 ml per consecutive day. The patient was followed up at 1 and 6 months after surgery in the clinic or by phone for parathyroid function, swallowing function, cosmetic satisfaction, neck sensation and movement, etc. The swallowing function was assessed using Swallowing Impairment Index-6 (SIS-6) comprising six questions reported by Lombardi (8). The Voice Handicap Index-10 (VHI-10) reported by Deary was used for subjective voice outcome analysis (9). The VHI-10 reliably identifies abnormal voice when a score is greater than 11 (10). The cosmetic results were measured by a 10-point scale with clinical evaluation: 0–3, fair cosmetic results; 4–6, good cosmetic results; and 7–10, excellent cosmetic results. Neck sensation and movement dysfunctions were surveyed by questionnaire with a 4-point scale, using a reference period: 0, none; 1, mild; 2, moderate; and 3, severe.

### Data Analysis

Mean ± SD or median ± interquartile range (IQR) were used for continuous variables depending on their distribution. Categorical variables were described as frequency (percentage). For continuous variables, we used the Kolmogorov–Smirnov test to analyze the normalization of the distributed data and used the Mann–Whitney U tests to analyze non-normally distributed data. For categorical variables, a chi-square test and Fisher’s exact test were used for analysis. Kruskal–Wallis H test was used to compare the clinical score between two groups. p-Value <0.05 was taken as statistically significant.

## Results

**Figure 2:**
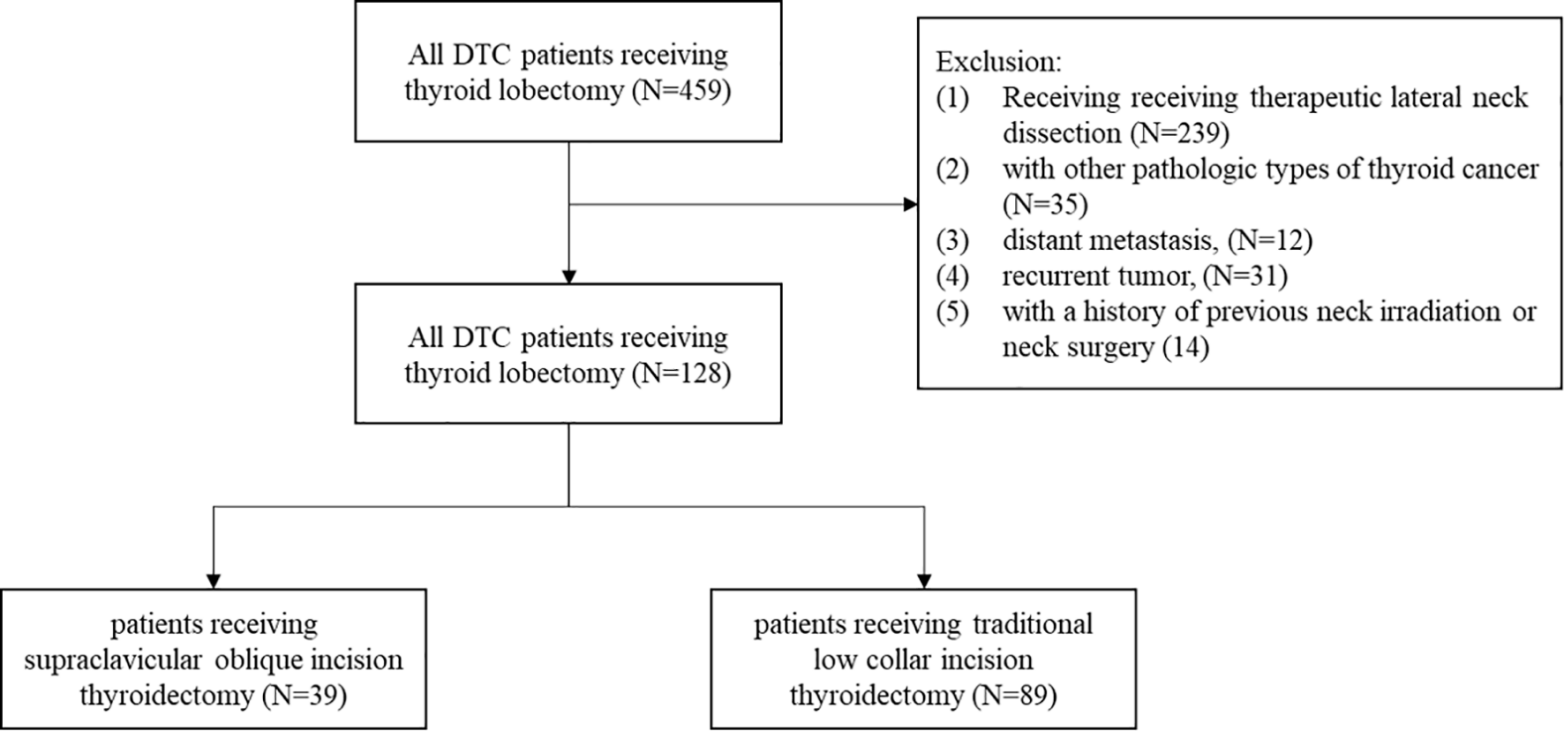
Flowchart of patients with DTC receiving thyroid lobectomy in the study. DTC, differentiated thyroid cancer.

After screening a total of 482 patients admitted to our center from October 2020 to October 2021, we finally included 128 patients in this study (as shown in **Figure 2**). Thirty-eight patients (30.5%) were operated on with SOIT and 89 patients (69.5%) with TLCIT.

### Comparison of Baseline Clinicopathologic Characteristics and Surgical Variables Between Supraclavicular Oblique Incision Thyroidectomy and Traditional Low Collar Incision Thyroidectomy Groups

The baseline clinicopathologic characteristics and surgical variables are presented in **Table 1**. For the demographic characteristics, there was no significant difference in age (38.7 ± 9.7 vs. 41.4 ± 11.4, year, p = 0.177), gender (7:32 vs. 27:62, p = 0.144), or body mass index (BMI) (23.5 ± 3.3 vs. 24.2 ± 3.1, kg/m^2^, p = 0.234) between the two groups. For the thyroid features, no significant difference was found in capsular extension (56.4% vs. 41.7%, p = 0.622), tumor size (0.7 ± 0.3 vs. 0.9 ± 0.4, p = 0.059), multifocality (5.1% vs. 14.6%, p = 0.216), or the presence of Hashimoto’s thyroiditis (15.4% vs. 20.5%, p = 0.501). Compared with the TLCIT group, the patients in the SOIT group were operated on with similar operative time (61.9 ± 12.1 vs. 59.9 ± 15.0, p = 0.425) but shorter and smaller incisions (4.4 ± 0.7 vs. 5.0 ± 1.0, p = 0.002). The number of the harvested LNs and the cancer-positive LNs found during operation between the two groups showed no significant difference. Moreover, in terms of postoperative management, the SOIT group was found with shorter duration of postoperative drainage (2.4 ± 0.5 vs. 2.7 ± 0.9, p = 0.019), less volume of postoperative drainage (48.4 ± 24.6 vs. 60.3 ± 22.8, p = 0.040), and shorter postoperative hospitalization (3.2 ± 0.5 vs. 3.6 ± 0.9, p = 0.006).

**Table 1:** The clinicopathologic characteristics and surgical variables of SOIT and TLCIT groups.

Characteristics	SOIT group N = 39	TLCIT group N = 89	p-Value
Age (years)	38.7 ± 9.7	41.4 ± 11.4	0.177
Male:female	7:32	27:62	0.144
Body mass index (kg/m^2^)	23.5 ± 3.3	24.2 ± 3.1	0.234
Capsular extension	22 (56.4)	46 (51.7)	0.622
Tumor subtype			
Tumor size, cm	0.7 ± 0.3	0.9 ± 0.4	0.059
Multifocality	2 (5.1)	13 (14.6)	0.216
Hashimoto’s thyroiditis	6 (15.4)	18 (20.5)	0.501
Operative time (min)	61.9 ± 12.1	59.9 ± 15.0	0.425
Incision length (cm)	4.4 ± 0.7	5.0 ± 1.0	0.002
Harvested LNs	1.9 ± 2.1	2.9 ± 2.7	0.024
Positive LNs	0.5 ± 1.1	0.6 ± 1.0	0.733
Duration of postoperative drainage (ml)	2.4 ± 0.5	2.7 ± 0.9	0.019
Postoperative hospital days	3.2 ± 0.5	3.6 ± 0.9	0.006
Volume of postoperative drainage (ml)	48.4 ± 24.6	60.3 ± 22.8	0.040

LNs, lymph nodes; SOIT, supraclavicular oblique incision thyroidectomy; TLCIT, traditional low collar incision thyroidectomy.

### The Follow-Up Outcomes of Supraclavicular Oblique Incision Thyroidectomy and Traditional Low Collar Incision Thyroidectomy

The results of 1-month and 6-month follow-ups are shown in **Table 2**. At 1 month after the operation, the patients in the SOIT group had a significantly lower incidence of hypoparathyroidism (17.9% vs. 37.5%, p = 0.029). For the self-perception of the patients, the SOIT group showed a higher cosmetic satisfaction score (9.1 ± 1.7 vs. 8.4 ± 2.0, p = 0.036), a lower percent of abnormal neck sensation (p = 0.010), and a lower percent of abnormal neck movement (p = 0.142). Although the SOIT group had a higher VHI-10 score than that of the TLCIT group, statistically, it was not significant (p = 0.259). At 6 months after the operation, the SOIT group was still found with a higher cosmetic score (9.3 ± 1.1 vs. 8.5 ± 1.4, p < 0.001), a lower percentage of abnormal neck sensation (p = 0.031), and a lower percentage of abnormal neck movement (p = 0.005). Meanwhile, the percent of hypoparathyroidism (7.7% vs. 11.4%, p = 0.755) and the VHI-10 score (p = 0.333) showed no significant difference between the two groups.

**Table 2:** The comparison of 1-month and 6-month follow-up outcomes in SOIT and TLCIT groups.

Variables	1 month after surgery			6 months after surgery		
	SOIT group N = 39	TLCIT group N = 89	p-Value	SOIT group N = 39	TLCIT group N = 89	p-Value
Hypoparathyroidism	7 (17.9)	33 (37.5)	0.029	3 (7.7)	10 (11.4)	0.755
Cosmetic scores	9.1 ± 1.7	8.4 ± 2.0	0.036	9.3 ± 1.1	8.5 ± 1.4	<0.001
Abnormal neck sensation			0.010			0.031
0	16 (12.1)	22 (17.3)		23 (18.1)	36 (28.3)	
1	20 (15.7)	43 (33.9)		16 (12.6)	43 (35.4)	
2	3 (2.4)	15 (11.8)		0 (0)	7 (5.5)	
3	0 (0)	8 (6.3)		0 (0)	0 (0)	
Abnormal neck movement			0.142			0.005
0	29 (74.4)	54 (61.4)		36 (92.3)	61 (15)	
1	7 (17.9)	21 (23.9)		2 (5.1)	15 (17.0)	
2	2 (5.1)	9 (10.2)		1 (2.6)	11 (12.5)	
3	1 (2.6)	4 (4.5)		0 (0)	1 (1.1)	
VHI-10 score			0.259			0.333
>11	7 (17.9)	24 (23.5)		1 (2.6)	6 (6.8)	
≤11	32 (82.1)	64 (76.5)		28 (97.4)	82 (93.2)	

VHI-10, Voice Handicap Index-10; SOIT, supraclavicular oblique incision thyroidectomy; TLCIT, traditional low collar incision thyroidectomy.

## Discussion

Thyroid surgery is the mainstay of treatment for thyroid tumors, which targets complete removal of the thyroid lesion without damaging the surrounding vital tissues (11). Traditional thyroid surgery by low collar incision approach usually involves a long incision and may cause postoperative discomfort such as swallowing dysfunction and swelling in the free flap area. To better satisfy the cosmetic and other life-quality demands of patients, various surgical methods have been developed (12).

One surgical approach development is accessing the thyroid compartment through the creation of a subcutaneous tunnel from the chest wall, axilla, or other positions using endoscopy, hence leaving no scar on the neck (13–15). However, the creation of extensive subcutaneous tunnels may lead to postoperative localized scar growth, and patients are prone to discomfort such as skin tugging. Another is minimally invasive surgery, including small incision thyroidectomy with or without endoscopic assistance (16, 17). However, this approach has strict indications and a probability of conversion to the TLCI approach. It was reported that minimally invasive surgery can be applied to 20%–30% of patients who need thyroidectomy (18, 19).

In our center, SOIT, which was first reported by Chen (7), has been performed since 2018. However, the safety and efficacy of this incision were yet to be confirmed due to a small number of related studies. In this retrospective study, we found that the patients who received SOIT had a smaller incision, shorter duration of postoperative drainage, a smaller volume of postoperative drainage, and shorter postoperative hospitalization, as compared with those who received TLCIT. At 1-month and 6-month follow-ups, the SOIT group also showed better patient-related outcomes, including voice assessment, swallowing function, cosmetic satisfaction, and neck sensation and movement. These advantages over TLCIT may benefit from the following characteristics of SOIT summarized in our center during a 3-year practice: 1) the surgical field can be accessed by the natural anatomical gap between neck muscles without severing the anterior cervical band; 2) it is easier and safer to perform dissection and ligation under direct visual inspection, especially for the upper pole of the thyroid; 3) the parathyroid glands and important vessels in the neck, such as the internal jugular vein, can be identified more accurately without accidental removal or injury; 4) the postoperative neck tissue damage reaction and scar adhesions are milder than those caused by TLCIT.

It is widely accepted that LN yield (LNY) can reflect the extent and quality of central neck dissection (20, 21). The LNY (unilateral) and positive LN number in all included patients are (expressed by median (IQR)) 1.0 (0, 3.0) and 0 (0, 1.0) respectively. It seems that LNY in this study is a smaller number compared with that in previous studies (20–22). We consider that there may be several reasons: the patients included in this study were planned to undergo thyroid lobectomy without the preoperative ultrasonography evidence of positive LN. In these patients, central compartment neck dissection (CND) was performed prophylactically rather than therapeutically based on the current guideline (3). It is reported that the LNY is significantly lower in prophylactic CND than in therapeutic CND (1), as clearing the central compartment LN tends to be rather conservative in prophylactic CND. Nevertheless, for LNY and the positive LN, no significant difference was found between the SOIT and TLCIT groups.

According to our practice and studies reported before, the indications of SOIT should be limited to patients with the following characteristics: with low-to-intermediate risk, well DTC of a diameter < 4 cm, and a thyroid gland volume < 30 cc (19, 23). On the other hand, the patients with a history of thyroiditis, previous neck surgery or head/neck irradiation, palpable lymphadenopathy, large goiters, or aggressive and poorly DTCs are not suitable for SOIT. Moreover, for bilateral thyroid lesions needing total thyroidectomy, it appeared that thyroidectomy by two-sided SOI was not easier or more effective than TLCIT in our 20 cases of practice. More studies are required to determine the efficacy of the two-sided SOI approach in patients who need total thyroidectomy.

Our research has two limitations. First, this single-center retrospective study enrolled only a small number of patients, and further research with larger sample sizes is needed. Second, some of the questionnaires, such as those for evaluating cosmetic satisfaction, and neck sensation and movement, are highly subjective and yet to be validated.

## Conclusion

Our study suggests that minimally invasive surgery using the SOI provides superior surgical and patient-related outcomes compared with conventional surgery using a TLCI for the treatment of DTC. SOIT is potentially a valuable alternative operative technique to TLCIT.

## Data Availability Statement

The raw data supporting the conclusions of this article will be made available by the authors, without undue reservation.

## Ethics Statement

The studies involving human participants were reviewed and approved by the institutional review board of a thyroid database (TDatabase) at the Affiliated Drum Tower Hospital of Nanjing University Medical School (No. 2021 GLTDMC-008). The patients/participants provided their written informed consent to participate in this study.

## Author Contributions

CQ and BJ: study design. CQ, BJ, and SS: acquisition of data and statistical analysis. CQ, BJ, CJ, and CZ: analysis and interpretation of data. CQ and CZ: drafting of the manuscript. SS, YL, and LS: critical revision of the manuscript. YL and LS: study supervision and guidance. All authors have read and approved the final version of this manuscript, including the authorship.

## Conflict of Interest

The authors declare that the research was conducted in the absence of any commercial or financial relationships that could be construed as a potential conflict of interest.

## Publisher’s Note

All claims expressed in this article are solely those of the authors and do not necessarily represent those of their affiliated organizations, or those of the publisher, the editors and the reviewers. Any product that may be evaluated in this article, or claim that may be made by its manufacturer, is not guaranteed or endorsed by the publisher.
